# Chimeric antigen receptor T-cell (CAR T-cell) and tumor-infiltrating lymphocytes (TILs) therapies in gastrointestinal malignancies: review of literature for clinical applications

**DOI:** 10.1007/s12032-025-03040-5

**Published:** 2025-09-20

**Authors:** Mohamed Saad Sayed, Ahmed Farid Gadelmawla, Osama Abouelenin, Elsayed S. Moubarak, Nada S. Jibril, Mahmoud Kandeel, Hebatullah Abdulazeem

**Affiliations:** 1 Faculty of Medicine, Beni-Suef University, Beni Suef, 62511 Egypt; 2 Faculty of Medicine, Menoufia University, Menoufia, Egypt; 3 Medical Research Group of Egypt (MRGE), Negida Academy, Arlington, MA USA; 4 Faculty of Medicine, Alexandria University, Alexandria, Egypt; 5 Faculty of Medicine, Cairo University, Cairo, Egypt; 6 Department of Biomedical Sciences, College of Veterinary Medicine, King Faisal University, 31982 Al-Ahsa, Saudi Arabia; 7 Department of Pharmacology, Faculty of Veterinary Medicine, Kafrelsheikh University, Kafrelsheikh, 33516 Egypt; 8 Chair of Epidemiology, TUM School of Medicine and Health, Technical University of Munich, Munich, Germany

**Keywords:** Chimeric antigen receptor T cells, CAR T-cells, Gastrointestinal malignancies, GI malignancies, Tumor-infiltrating lymphocytes, TIL therapy

## Abstract

Gastrointestinal malignancies (GI malignancies) have had a notoriously dismal prognosis throughout history. The primary therapeutic approaches to treat and manage GI malignancies are immunotherapy, radiotherapy, surgery, and chemotherapy, which may include monotherapy or a combination of these therapies to boost the effect. Nevertheless, the recurrence and metastasis rates remain elevated. In recent decades, immunotherapies have had a powerful impact when included in treatment regimens. In hematologic malignancy, chimeric antigen receptor T cells (CAR-T cell) have shown a promising anticancer impact as one of the immunotherapies. It gives a promising treatment option for solid tumors, including colorectal cancers. In recent clinical trials, the CAR-T cells showed a promising effect on pancreatic, colorectal, esophageal, hepatocellular, and gastric cancers. Tumor-infiltrating lymphocyte (TIL) therapy is another immunotherapy option with promising option for GI malignancies. Through the process of designing the TIL therapy, T cells are extracted and designed according to the nature of the GI malignancy. In this review, we addressed the clinical applications of both therapies while highlighting the challenges and possible strategies to overcome them. CAR T-cells and TIL therapies showed good responses with tolerable and acceptable side effects in treating GI malignancies such as pancreatic, colorectal, gastric, and hepatocellular cancers, while the immunosuppressive tumor microenvironment (TME) inhibiting the activity of immunotherapy and impeding its efficacy is a significant challenge.

## Background

The continuous rise in the prevalence of Gastrointestinal (GI) malignancies makes it a significant health problem threatening human life [1, 2]. GI malignancies, including esophageal, stomach, liver, pancreas, and colorectal cancers (CRC), represent more than 25% of all cancers worldwide [1, 2]. Esophageal cancer (EC) is a major GI malignancy [3, 4]. The GLOBOCAN database was used in 2020 to gather data on new cases and estimated that more than 600,000 new cases, with more than 500,000 related deaths, occurred [3, 4]. Based on the same database, the latest cases of gastric cancers (GCs) in 2020 were estimated at 1.1 million, with more than 700,00 related deaths [3, 5]. In 2020, at an alarming rate, the incidence of CRCs rose [6]. Based on the data from the GLOBOCAN database, the incidence of CRC was estimated at 1.9 million new cases, with 53,000 CRC-related deaths in 2024 [3, 6, 7]. Several risk factors, such as smoking, infections, obesity, dietary habits, and alcohol, are shared in most of the GI malignancies [1, 8]. In the presence of these factors, it is hypothesized that progressive changes in these risk factors may contribute to increasing the incidence of whole GI malignancies [9, 10].

The stomach and pancreatic cancer are diagnosed late, resulting in a delay in starting treatment, which leads to a poor prognosis for recovery [11]. The challenges in treating gastrointestinal tumors arise from late diagnosis, as well as resistance to existing conventional therapies, such as surgery, chemotherapy, and radiation, which cannot address the tumor microenvironment (TME) [12]. Also, the immunosuppressive nature of the complex TME constitutes a major barrier to effective treatment [12]. Immunotherapy has an effective role in cancer treatment, as it harnesses the body’s immune system, directs it towards malignant cells, and eliminates them. Unlike traditional chemotherapy, which randomly affects rapidly dividing cells, current immunotherapy strategies and approaches enhance the ability of the immune system to recognize and fight cancer cells [13].

Chimeric Antigen Receptor T-cell (CAR-T cell) therapy and Tumor-Infiltrating Lymphocytes (TILs) therapy are among the most promising methods in cancer immunotherapy [14, 15]. CAR-T cell therapy is a technique in which white blood cells, known as T cells, are genetically modified to become more efficient in recognizing and attacking cancer cells [16]. CAR-T cell therapy has achieved significant success in treating certain types of blood cancers. It is currently being investigated for the treatment of gastrointestinal cancers, as demonstrated in the study by Marofi et al [17]. The basic idea of TIL therapy is to recognize and extract immune cells already present within the tumor, where they are activated in the laboratory and then returned to the body to fight cancer more effectively. However, TIL therapy is distinguished by being customized for each patient based on the nature of their gastrointestinal system [17].

Despite significant progress in immunotherapy, the application of CAR-T cell therapies and TIL therapies in malignant tumors remains unclear and an area of research. The unique challenges TME poses require conducting a lot of clinical research to reach precise and proven results regarding these treatments [13]. CAR-T cell therapy in particular faces many obstacles, such as limited tumor penetration and extra-tumor T cell toxicity, which requires adequate research and addressing, so that the treatment can perform its role as effectively as possible [17].

The application of CAR-T cell and TIL therapies in gastrointestinal malignancies remains limited. We aim to review the current evidence about CAR-T cell therapy and TIL therapies in GI malignancies by addressing the results of available clinical trials and clarifying the mechanism of action and possible limitations in the clinical applications.

## Overview of adoptive cell therapies

**Fig. 1:**
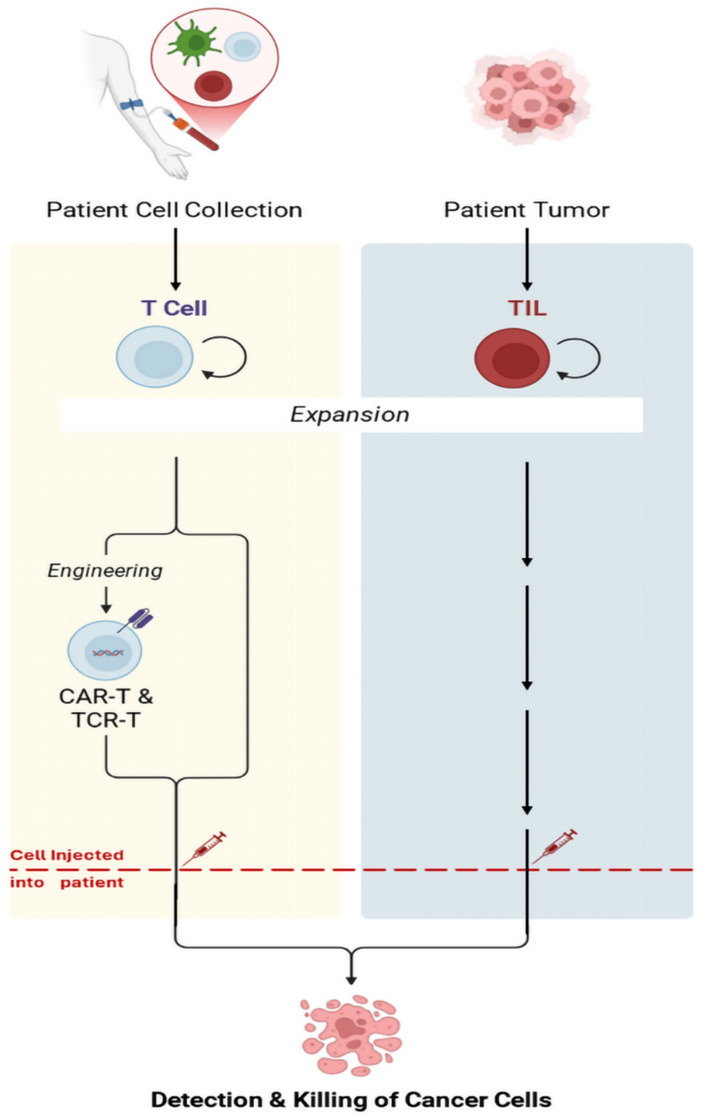
Overview of adoptive cell therapy (ACT) Strategies in Cancer Treatment. This figure was adapted from the paper “In vivo gene editing and in situ generation of chimeric antigen receptor cells for next-generation cancer immunotherapy [19]” and relied on the biorender template: https://app.biorender.com/biorender-templates/t-667f10a092a4150389a44080

Adoptive cell therapy (ACT) is a naturally occurring therapeutic approach that effectively fights cancer, as it modifies or expands existing immune cells outside the body before reinjecting them into the patient [18]. It includes many modalities, including CAR-T cell therapies, TIL therapy, and engineered T-cell therapy with T-cell receptors (TCR-T) as illustrated in Fig. 1.

CAR-T therapy involves genetically modifying T cells to express synthetic receptors (CARs) [15, 20, 21]. These receptors recognize specific tumor antigens, stimulating the immune system to respond [15, 20, 21]. CAR-T’s remarkable and successful role in hematologic malignancies such as acute lymphoblastic leukemia (ALL) was demonstrated in the Staudt et al. study [22]. This has prompted the US Food and Drug Administration (FDA) to approve therapies for cancer [22]. However, its application in solid tumors, including gastrointestinal cancers, faces challenges such as poor tumor infiltration and TME [22].

On the other hand, TIL therapy relies primarily on isolating naturally occurring tumor-reactive lymphocytes from tumor tissue, expanding them outside the body, and then reinjecting them into the patient’s body [12]. It has shown effective results in treating immune tumors such as melanoma, and research continues into its role in treating gastrointestinal cancers [12].

TCR-T therapy is a form of ACT that genetically engineers T cells to express TCRs that recognize tumor-associated antigens via major histocompatibility complex (MHC) molecules [17]. Unlike CAR-T cells, which target antigens outside the body, TCR-T cells recognize tumor antigens inside cells [23]. This method increases the range of malignancies that can be targeted, which improves the cure rate [24]. Despite all the above, these CAR-T cells require compatibility with the major histocompatibility complex, which impacts their spread [17].

The mechanism of action of each drug varies from one method to another, depending on the type of treatment. By the start of CAR-T therapy, T cells are extracted from the patient, genetically modified to express CARs, and then reinjected to directly attack tumor cells [24]. TIL treatment enhances the body’s immune response by collecting lymphocytes that infiltrate the tumor and then reinjecting them to make their effect stronger than the tumor itself [25]. So, in conclusion, the TCR-T therapy works similarly to CAR-T, but the difference lies in the ability of TCR-T to recognize antigens inside cells, which makes it capable of treating some solid tumors [17].

Despite the potential and success of T-cell therapy, it faces many obstacles, such as immunosuppression via the TME and limited stability of T cells [26, 27]. Current explorations have shown that improving CAR designs and T-cell therapy protocols, where T-cell therapy is combined with ICIs, enhances its effectiveness and reduces the rates of limitations [11]. Given the continuous progress in stem cell therapy, it represents a single front and an effective treatment in combating malignant tumors in the digestive system. Therefore, the research focus must continue to solve the current limitations of medications to prove their effectiveness and improve patient outcomes [12].

## CAR T-cell therapy in GI malignancies

### Mechanism of action

**Fig. 2:**
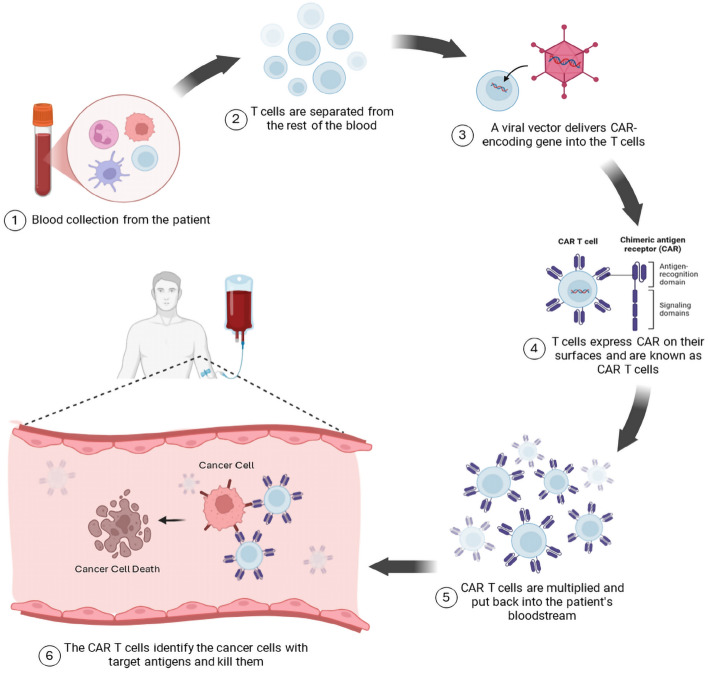
Generation and mechanism of action of CAR T-cell in GI malignancy. This figure was adapted from the previous paper, “Inflammation, Infiltration, and Evasion—Tumor Promotion in the Aging Breast [33]” with license: *CC BY 4.0* [33]

Advancements in knowledge regarding the composition and function of T-cell-mediated immunity and gene therapy enabled genetic reprogramming of T-cells, thus presenting T-cells that are optimized for better recognition of tumor antigens, cell survival, and expansion, known as CAR-T cells [28]. One of the earlier models developed for CAR T-cells, which was regarded later as the first-generation CAR-T cell therapy, was described by Eshhar et al. in 1993 [29]. This first generation introduced new receptors, formed of a variable single-chained fragment that recognizes a specific antigen on the cancer cell, a hinge region for flexibility, a transmembrane domain anchoring the CAR to the cell membrane, and an intracellular domain for T cell activation [30]. This modification enabled CAR-T cells to target tumor-specific antigens. It enabled the CAR-T cells to target glycolipid, carbohydrate, and protein antigens, making them superior to the non-modified T-cells that can only recognize protein antigens, as illustrated in Fig. 2 [31]. Moreover, CAR-T cells could bind directly to these antigens without needing antigen-presenting cells [32].

However, this first generation showed poor anti-tumor effects as it could not maximally activate other unprimed naive T-cells owing to the absence of costimulatory domains [34, 35]. Costimulatory domains are found on the surface of T-cells, and the costimulatory signals are responsible for enhancing proliferation, survival, cytotoxic function, and memory formation [36]. As a result, second-generation CAR T-cell therapy was developed, and the costimulatory domains were added to the receptor construct [37]. Thus, the CAR-T cell immunotherapy could prime and amplify, resulting in a better immunological response to malignant cells. CAR-T cells' most common costimulatory domains are derived from CD28 or 4-1BB [38]. Third-generation CAR-T cell therapy involves providing additional costimulatory domains to the receptor, including two or more costimulatory domains [28]. The fourth-generation CAR-T cell therapy involves a cytokine-inducible gene to recruit other immune cells and manipulate the tumor microenvironment [30]. The fifth and latest generation, known as the next generation, has intracellular cytokine receptor fragments associated with activating a transcription factor, promoting CAR-T cell proliferation and safety [38, 39].

### Targets in GI cancers

According to the latest Global Burden of Disease study, the incidence of GI cancer was 5.26 million cases, with 3.7 million deaths [40]. CRC was the most common GI malignancies, followed by gastric, pancreatic, hepatic, and biliary cancers [40]. CAR-T cell therapy is utilized for GI cancers by targeting GI cancer antigens, such as carcinoembryonic antigen (CEA), mucin-1 (MUC1), alpha-fetoprotein (AFP), human EGFR 2 (HER2), Claudin18.2 (CLDN 18.2), and glypican-3 (GPC3) [41]. Most tumor antigenic targets lack specificity for one GI cancer. Based on antigen expression patterns, tumor antigens are classified as either tumor-specific antigens (TSAs) that are only found in tumors or tumor-associated antigens (TAAs) that could also be present in normal tissues. When CAR-T cells target TAAs, they could attack normal tissues, resulting in on-target, off-tumor toxicity, which occurs owing to their low specificity, and this effect could be fatal [42]. Moreover, there are heterogeneities in tumor antigen expression, either in the quantity or the type of antigen expressed within the same tumor, and for primary and recurrent lesions [43]. As CAR-T cell therapy is developed to target a single antigen, some malignant cells could escape CAR-T cells, thus limiting their efficacy and increasing the risk of recurrence [17].

### Clinical applications

The representative clinical trials of CAR-T cell therapies for patients with GI cancers are summarized in Table 1. This table shows the available trials with the target, cancer type, no. of patients, efficacy, and safety outcomes.

**Table 1:** Clinical trials of CAR-T cells for patients with GI cancers

Author name, country, year	Phase	Target	Cancer type	Patients No.	Efficacy		Major toxicities reported	
					ORR	mPFS, months	Any grade, %	≥ Grade 3, %
Pancreatic cancers (PC)								
Liu et al., China (2020) [44]	Phase 1	EGFR	PC	16	28%	3.0	Mucositis oral (43%), dry skin (43%) Pleural effusion (31%)	Dermatitis herpetiformis (13%) Pleural effusion (13%)
Feng et al., China (2018) [45]	Phase 1	HER2	PC/BTC	11	9%	4.8	Fever/chill (100%), fatigue (36%), transaminase elevation (18%)	Fever/chill (9%) Transaminase elevation (9%)
Guo et al., China (2018) [46]	Phase 1	EGFR	BTC	19	6%	4.0	Fever/chill (100%), fatigue (84%), ascites/pleural effusion (21%), nausea/vomiting (16%)	Ascites/pleural effusion (16%)
Wang et al., China (2018) [47]	Phase 1	CD133	Multiple malignancies	23 (14 with HCC, 7 with PC, and 2 with CRC)	13%	5 (7 in HCC)	Leukopenia (34.7%)	NR
Beatty et al., US (2018) [48]	Phase 1	Mesothelin	PC	6	0%	NR	CRS (0%), ICANS (0%)	NR
Hepatocellular carcinoma (HCC)								
Nakajima et al., Japan/US (2024) [49]	Phase 1	GPC3	HCC/GC/liposarcoma	11	0%	NR	Nausea (55%), CRS (55%)	GI toxicities (0%), CRS (0%)
Zhang et al., China (2024) [50]	Phase 1	GPC3	HCC	24	56.5%	NR	CRS (91.7%)	CRS (4.2%)
Shi et al., China/United States (2020) [51]	Phase 1	GPC3	HCC	13	15%	NR	Decreased appetite (38%), CRS (69%), fever (100%), chills (54%)	Hyperbilirubinemia (15%), CRS (7%, grade 5)
Dai et al., China (2020) [52]	open-label, phase II trial	CD133	HCC	21	5%	6.8	Hyperbilirubinemia (19%)f Nausea (14%)f	Hyperbilirubinemia (19%) Nausea (0%)
Colorectal cancer (CRC)								
Qi et al., China (2024) [53]	Phase 1	GUCY2C	CRC	20 (high dose level: 13)	26% (high dose level: 39%)	3.0 (7.0)	Diarrhea (70%), elevated T-Bil (75%), CRS (85%)	Diarrhea (55%), hyperbilirubinemia (15%), CRS (5%)
Chen et al., China (2024) [54]	Phase 1	CD19/ GUCY2C	CRC	15	40%	Dose level l: 3, dose level 2: 6	Diarrhea (93%), CRS (87%), nausea (40%)	Diarrhea (40%) CRS (0%)
Zhang et al., China (2017) [55]	Phase 1	CEA	CRC	9	0%	NR	Fever (22%)	NR
Gastric, esophageal cancers, and GEJC								
Luo et al., China (2024) [56]	Open-label phase 1	EpCAM	GC	11	30%	NR	Abnormal liver function (36%), CRS (45%)	Abnormal liver function (27%), CRS (27%)
Botta et al., United States (2024) [57]	Phase 1b	CLDN18.2	GEJC/GC/PC	19	GC: 43% PC: 17%	GC: 5.7, PC: 2.7	Vomiting (16%) CRS (90%)	Vomiting (0%) CRS (11%)
Qi et al., China (2024) [58]	Phase 1	CLDN18.2	GC/GEJC Other GI cancers	98 (Cohort 1: 61^*^)	39% (Cohort 1: 36%)	4.4 (Cohort 1: 4.2)	Nausea (67%), vomiting (53%), CRS (97%)	Nausea (1%), vomiting (3%), CRS (0%)
Qin et al., China (2024) [59]	Phase 1a	P329G	GEJC/GC/PC (CLDN18.2-positive)	5	25%	NR	Decreased appetite (80%), vomiting (40%), CRS (0%), ICANS (0%)	Decreased appetite (20%)
Mixed GI cancer patients								
Thistlethwaite et al., UK (2017) [60]	Phase 1	CEA CAM5	GI cancers	14	0%	NR	Respiratory toxicities	NR
Zhang et al., China (2024) [61]	Phase 1	CEA	Solid tumors (GI cancers)	40 (39)	I.P: 25%, I.V: 8%	I.P: 3.3, I.V: 3.1	I.P: CRS (69%), diarrhea/colitis (31%) I.V: CRS (79%), diarrhea/colitis (4%)	I.P: CRS (0%), diarrhea/colitis (25%) I.V: CRS (0%), diarrhea/colitis (4%)

*BTC* biliary tract cancer, *CAR-T* chimeric antigen receptor-T, *CEA* carcinoembryonic antigen, *CRC* colorectal cancer, *CRS* cytokine release syndrome, *GC* gastric cancer, *GEJC* gastroesophageal junction-cancer, *HCC* hepatocellular carcinoma, *HER2* human epidermal receptor 2, *ICANS* immune effector cell–associated neurotoxicity syndrome, *I.P* intraperitoneal transfusion, *IV* intravenous transfusion, *mPFS* median progression-free survival, *ORR* objective response rate, *PC* pancreatic cancer

*Cohort 1: dose expansion cohort satri-cell monotherapy

#### Pancreatic cancer

Multiple studies were conducted to investigate the use of CAR-T cell therapy for pancreatic cancers in both animal and human cell Lines, with few published phase 1 clinical trials [62]. Phase 1 trial investigated CAR-T cell therapy for the 23 metastatic GI cancer patients targeting mesothelin in these carcinomas; seven out of 23 were pancreatic cancer patients who were refractory to chemotherapy [63]. The clinical response to treatment was limited, with three out of five patients showing no response, and the treatment might be generally safe except for one case of toxicity [63]. Another phase 1 trial investigated CAR-T cell therapy targeting the cancer stem cell marker CD133 [47]. This trial involved repeated infusions of the CAR-T cells, and by the end of the study, three out of the seven pancreatic cancer patients maintained stable disease for a maximum of 10.25 months, two patients did not respond to therapy, and the remaining two patients showed partial remission for 2.4 months [47]. Two trials evaluated CAR-T cell targets against CLDN 18.2 and involved a single infusion. Both studies had five patients, of whom two and three patients had stable disease for each study, respectively [47, 64]. A recent trial involving 24 pancreatic cancer patients with metastasis used CAR-T cell therapy targeting CLDN 18.2; The treatment showed encouraging anticancer efficacy signals and a tolerable safety profile [65].

#### Hepatocellular carcinoma

GPC3 is a glycoprotein expressed in most hepatocellular carcinoma (HCC) patients with minimal expression in normal and cirrhotic liver tissue, making it a suitable target for CAR-T cell therapy [66]. A recent trial on advanced unresectable HCC patients using GPC3 CAR T-cells armored with TGF-b receptor II showed an encouraging antitumor response and good safety in those patients [50]. Moreover, they found tumor reductions in 90.9% of HCC patients for intra- and extrahepatic lesions [50]. These findings are similar to an earlier trial investigating autologous GPC3 CAR-T cell therapy in HCC patients with induced lymphodepletion; the patients had a survival rate of 50.3% by 6 months and 42% after one year [51]. AFP is a glycoprotein found in high concentrations in different malignancies, including HCC. Animal studies showed that CAR-T cells targeting AFP successfully decreased tumor growth and dissemination [67]. A recent animal study investigated dual-targeted CAR-T cell therapy for AFP and GPC3 [68]. This therapy showed better cytokine secretion and significant malignant cell-killing effects, highlighting its superiority to GPC3 or AFP single-targeted therapy. There are still no published trials investigating AFP-targeted CAR-T cell therapy for HCC patients; thus, more evidence is needed to conclude its safety and efficacy [69].

#### Colorectal cancers

CEA is a tumor marker expressed in GI malignancies, including colorectal cancers (CRCs). One trial investigated the safety and efficacy of escalating doses of CAR-T cells targeting CEA. This trial included ten patients; seven did not experience any severe adverse events, and seven achieved a stable disease state [55]. Natural killer group 2 member D ligands (NKG2D) are found on the surface of malignant cells, including CRC cells. CAR-T cells targeting NKG2D showed promising results, where six of nine patients treated with the highest dose levels achieved tumor control [70]. Another target for CRC is the epidermal growth factor receptor (EGFR). An in vitro study co-inoculating CAR-T cells targeting EGFR with miR-153 and tumor cells showed complete eradication of malignant cells. Yet, no published trials confirm their safety and efficacy in humans [71].

#### Gastric and esophageal cancers

CLDN 18.2 is expressed by different malignancies, including gastrointestinal cancers, making it a possible target for CAR-T cell therapy. A phase 1 open-label trial evaluated the safety and efficacy of CAR-T cells targeting CLDN18.2 in 98 patients with advanced gastrointestinal cancers; no treatment-related deaths, neurotoxicity, or dose-limiting toxicities were reported. The total disease control rate was 91.8% with a median progression-free survival of 4.4 months [58]. A case report of a patient with metastatic gastric cancer treated with CLDN 18.2-targeted T-cells had similar findings; the patient had previously progressed on multiple lines of combined immunotherapy, chemotherapy, and systemic therapy [72]. After two cycles of autologous CAR-T cell therapy, the patient showed a complete response to the target lesion with an improved quality of life and no severe side effects [72]. Another possible target for gastric cancer is HER2. A previous study on xenograft models investigating CAR-T cell therapy targeting HER2 on gastric cancer cells showed improved tumor inhibition and long-term survival, suggesting a need for clinical trials investigating the potential role of CAR-T cell therapy in HER-positive gastric cancer patients [73]. Esophageal cancers also express HER2. A preclinical study investigating CAR-T cell therapy targeting HER2 that included a CD28 costimulatory domain and a PD-1 blocking domain showed limited cytokine production, suggesting a low risk of systemic toxicity and off-tumor leakage [74]. This is similar to the findings of a recent review of preclinical studies indicating promising antitumor effects of HER2-targeting CAR-T cell therapy against esophageal cancers both in vivo and in vitro [75].

### Challenges and limitations

**Fig. 3:**
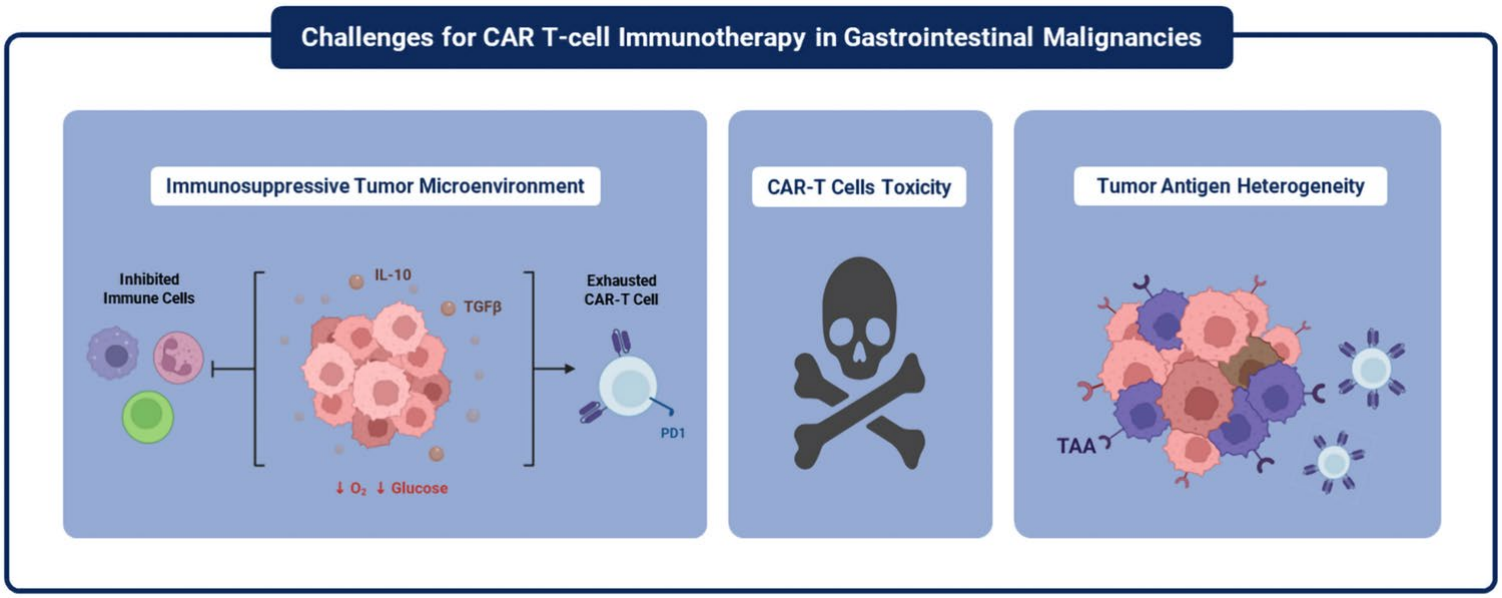
Challenges for CAR-T cell immunotherapy in GI Malignancies. This figure was adapted from the papers “Therapeutic potential of CAR T cell in malignancies: A scoping review [84]”, and “Adoptive cellular therapy in solid tumor malignancies: review of the literature and challenges ahead [85]” under license: CC BY 4.0 [84, 85]

One challenge to the efficacy of CAR-T cell therapy is the immunosuppressive TME that was shown to impair T-cell metabolism and function [76, 77]. The TME is characterized by elevated lactates due to glycolytic metabolism, hypoxia, and high concentrations of suppressive metabolites and reactive oxygen species, as illustrated in Fig. 3 [76, 77]. Besides malignant cells, the TME contains many cells such as tumor-associated macrophages, fibroblasts, and myeloid-derived suppressor cells [78]. These cells modify the extracellular matrix, create an immunosuppressive TME, and augment tumor development through different mechanisms involving releasing growth factors, cytokines, and chemokines; producing extracellular proteins; and forming an inflammatory [79, 80]. Promotion of CAR-T cell migration to their target is important, especially for solid GI malignancies. Recent evidence showed better migration and long-term antitumor activity with TGF-β blocking and photothermal therapy [81]. Another technique to improve migration in solid tumors with hostile TME includes the use of inverted cytokine receptors to convert inhibitory signals into stimulatory ones [82]. Another technique to promote immune cell infiltration and activation in solid cancers includes adding engineered bacteria to the TME, thus facilitating the infiltration and activation of various immune cells, resulting in enhancing the overall immune response against tumors [83]. Some Limitations were noticed through the Literature regarding the CAR-T cell therapies studies; most of the available studies were phase 1 clinical trials, which lack randomization and the presence of baseline differences among the patients included. Also, the evidence was carried out through a small number of patients in each study alone; hence, the sample size ranged from 5 to 40 patients in most of the trials, except one trial included 98 patients with GC/GEJC, Other GI cancers [58].

Another challenge is that releasing cytokines associated with CAR-T cell therapy could reach toxic levels, leading to the manifestations of fever, muscle pain, and weakness, and could develop into systemic organ failure [86, 87]. The systemic increase in cytokines could be associated with neurotoxicity with a disrupted blood–brain barrier and increased cytokine levels within the cerebrospinal fluid, manifesting in headaches, delirium, seizures, or aphasia [88, 89]. Another challenge associated with the traditional CAR-T cells is their limited ability to recognize tumor cells, as they can only recognize one tumor surface marker, so the tumor cells lacking or poorly expressing it will escape these traditional CAR-T cells, making treatment more challenging [90].

### Ongoing trials

A limited number of published trials assessing CAR-T cell therapy in GI malignancies, with most evidence obtained from preclinical in vitro and in vivo studies, is available. Also, multiple ongoing trials assess CAR-T cell therapies in different GI malignancies. Table 2 shows the 30 ongoing and five complete trials evaluating CAR-T cell therapy exclusively in GI malignancies, all in phase I or II. There are variations in CAR targets and the underlying GI malignancies across the trials.

**Table 2:** Ongoing and completed trials for CAR T-cell therapy conducted in patients with GI malignancies

NCT number	Phases	Study status	GI malignancy	Intervention	Target	Primary outcome measures	Start date
NCT06353152	I	Recruiting	Gastric and gastroesophageal cancers	Claudin18.2-Targeted CAR-T	CLDN 18.2	Incidence of dose-limiting toxicity (DLT), treatment-emergent adverse events (AEs), Adverse Events of Special Interest (AESIs), and Serious Adverse Events (SAEs)	11/17/2023
NCT05538195	I/II	Recruiting	Gastric cancer, esophageal cancer, pancreatic cancer, CRC	CEA-targeted CAR-T	CEA	Incidence of AE and DLT	6/7/2022
NCT04404595	I/II	Active, not recruiting	Gastric cancer, pancreatic cancer	CT041	CLDN 18.2	Incidence of AEs and DLTs. Efficacy of CT041	10/23/2020
NCT05393986	I	Recruiting	Gastric cancer, pancreatic cancer, gastroesophageal Junction adenocarcinoma	CT048	CLDN 18.2	DLT, safety, and maximum tolerated dose	8/4/2022
NCT05028933	I	Recruiting	HCC, CRC, gastric cancer, pancreatic cancer	EPCAM CAR-T	CEA	DLT, safety, tolerability and AEs	9/30/2021
NCT05415475	I	Recruiting	Esophageal Cancer, stomach Cancer, pancreatic Cancer, CRC	CEA CAR-T	CEA	DLT, safety, tolerability and AEs	9/10/2021
NCT05396300	I	Active, not recruiting	Esophageal Cancer, stomach Cancer, pancreatic Cancer, CRC	CEA CAR-T	CEA	DLT, safety, tolerability and AEs	5/25/2022
NCT06857786	I	Not yet recruiting	Gastroesophageal Junction Adenocarcinoma, gastric Adenocarcinoma	CT041	CLDN 18.2	Incidence, type, and severity of AEs, AESIs and SAEs	3/10/2025
NCT06821048	I	Recruiting	Stomach cancer, esophageal cancer, pancreatic cancer, CRC	CEA-targeted CAR-T	CEA	Maximum tolerated dose, DLT, and incidence of AEs	7/24/2024
NCT05539430	I	Recruiting	Gastric cancer, gastroesophageal-junction-cancer, esophageal cancer, pancreatic cancer	LB1908	CLDN 18.2	Determining the optimal dose and assessing for safety and tolerability	4/18/2023
NCT02850536	I	Completed	Liver metastases	Anti-CEA CAR-T	CEA	Determining the safety and DLT	2/1/2017
NCT06134960	I	Not yet recruiting	Gastric cancer, pancreatic cancer	KD-496	NKG2D/CLDN18.2	Incidence of AEs, SAEs and dose-limiting toxicity	11/16/2023
NCT05319314	I	Recruiting	CRC	GCC19 CAR-T	Guanylate cyclase-C (GCC)	Incidence of AEs and DLT	8/1/2022
NCT06718738	I	Recruiting	CRC	IM96 CAR-T	Guanylyl cyclase 2C (GUCY2C)	Incidence of AEs and SAEs	12/15/2024
NCT05089266	I	Recruiting	CRC	MSLN CAR-T	Mesothelin	DLT and safety	11/30/2021
NCT05240950	I	Recruiting	CRC, metastatic liver cancer	Anti-CEA CAR-T	CEA	Incidence and severity of AEs	8/25/2022
NCT06653010	Early I	Recruiting	CRC	Universal CAR-T	GCC	Incidence of AEs, the maximum tolerated dose	10/23/2024
NCT06675513	Early I	Not yet recruiting	CRC	Armored GCC targeting WD-01 CAR-T	GCC	Incidence of AEs, the maximum tolerated dose	6/1/2025
NCT05759728	I/II	Recruiting	Metastatic CRC	CNA3103	LGR5	Incidence of AEs	10/24/2023
NCT05911217	I	Recruiting	Pancreatic cancer	CT041	CLDN 18.2	Disease-free survival	7/11/2023
NCT05779917	I	Recruiting	Pancreatic cancer	Mesothelin/GPC3/GUCY2C-CAR-T	Mesothelin/GPC3/GUCY2C	Incidence of DLT	3/10/2023
NCT06196658	Early I	Not yet recruiting	Pancreatic cancer, biliary cancer	Anti-EX02 CAR-T	EX02	Incidence and severity of AEs	1/1/2024
NCT06158139	I	Recruiting	Pancreatic cancer	CAR-T Cells Targeting B7-H3	B7-H3	Incidence of AEs	7/18/2024
NCT01897415	I	Completed	Pancreatic cancer	Anti-mesothelin CAR-T	Mesothelin	Incidence of AEs	2013-07
NCT03980288	I	Completed	HCC	CAR-GPC3 T Cells	Glypican-3 (GPC3)	Safety and tolerability, DLT and maximum tolerated dose	7/23/2019
NCT06461624	I	Recruiting	HCC	Anti-GPC3 CAR-T	GPC3	Incidence of DLT, AEs and SAEs; determining the maximum tolerated dose	7/1/2024
NCT06084884	I/II	Recruiting	HCC	AZD5851	GPC3	Incidence of AEs, SAEs and DLT	12/14/2023
NCT05783570	I	Recruiting	HCC	EU307 CAR-T	GPC3	Incidence and severity of AEs	8/24/2023
NCT05652920	I/II	Recruiting	HCC	Ori-C101	GPC3	Identification of the maximum tolerated dose	12/15/2022
NCT06590246	I/II	Not yet recruiting	HCC	Armored and GPC3-targeted autologous CAR-T	GPC3	Incidence of AEs and DLT	9/30/2024
NCT03884751	I	Completed	HCC	GPC3 CAR-T	GPC3	DLT, Safety, and tolerability	8/15/2019
NCT02395250	I	Completed	HCC	Anti-GPC3 CAR-T	GPC3	Incidence of AEs	2015-03
NCT06144385	I	Recruiting	HCC	GPC3 CAR-T	GPC3	Incidence and severity of AEs and DLT	3/24/2022
NCT06676982	I	Not yet recruiting	HCC	Anti-CD19 CAR-T	CD19	Incidence of AEs and DLT	1/7/2025
NCT06560827	I	Recruiting	HCC	CT011 CAR-GPC3 T Cells	GPC3	Incidence and severity of AEs	10/8/2023

## TIL therapy in GI malignancies

One promising treatment option for GI cancers is TIL therapy. This therapy uses lymphocytes that have naturally entered tumor tissues to strengthen the body’s immune system. Recent developments have clarified the mechanisms of action, therapeutic applications, difficulties, and current research.

### Mechanism of action

**Fig. 4:**
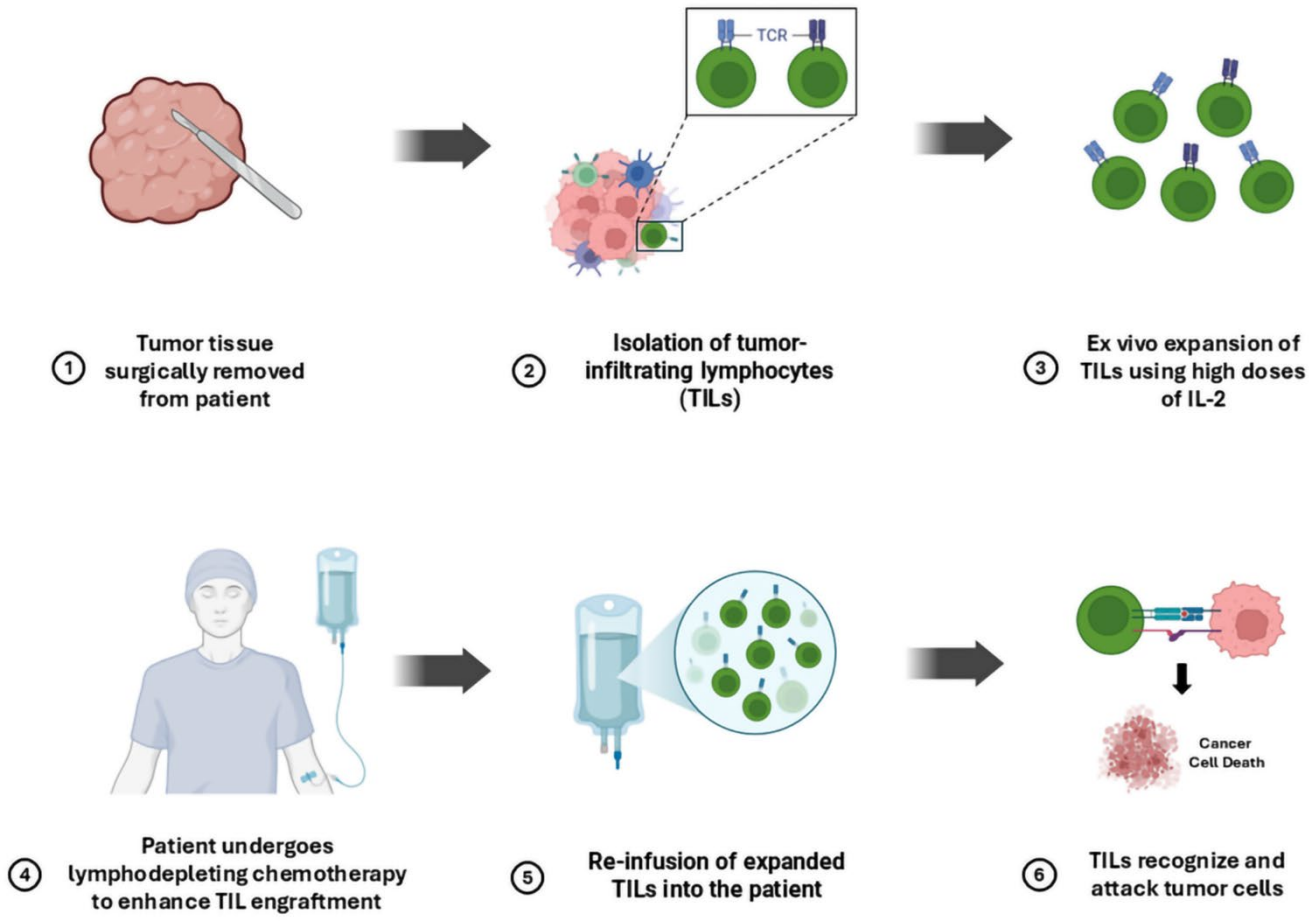
Mechanism of action of TIL therapy in GI malignancies

To target cancer cells, TIL therapy involves extracting lymphocytes from tumor tissues, growing them outside the body, and reintroducing them into the patient, as illustrated in Fig. 4. At the start of TIL therapy extraction, the lymphocytes are retrieved through surgical removal of tumor tissue from the patient, and then isolation of the TIL from the sample is done. Ex vivo expansion to promote the growth of these cells by adding large concentrations of interleukin-2 (IL-2) to their cultivation. The patients are exposed to a lymphocyte-depleting chemotherapy regimen to enhance TIL engraftment and effectiveness. Then the lymphocytes are reinfused into the patient when they have sufficiently expanded. This method uses TILs’ inherent ability to identify and target tumor cells, providing an individualized immunotherapeutic approach [91].

### Applications for GI cancers

TIL therapy has demonstrated promise in the treatment of gastrointestinal malignancies, especially those with characteristics of mismatch repair-deficient (dMMR) and microsatellite instability-high (MSI-H). These tumors frequently have a larger burden of mutations, which causes the production of neo-antigens that the immune system can recognize more easily. The effectiveness of TIL treatment for gastric and colorectal malignancies has been investigated in clinical trials. For example, a study showed that TIL treatment effectively treated patients with metastatic colorectal cancer who had MSI-H/dMMR, indicating its promise in this patient population [12, 92].

### Clinical applications

Several studies have investigated the application of TIL therapy across various GI malignancies:

#### Gastric cancer (GC)

A meta-analysis assessing the predictive significance of TIL therapy in GC evaluated different TIL subsets and their relationship to overall survival (OS) and disease-free survival (DFS) using 31 observational studies involving 4185 patients [93]. The findings demonstrated a strong correlation between increased survival and higher levels of CD8+ , FOXP3+ , CD3+ , CD57+ , CD20+ , CD45RO+ , Granzyme B+ , and T-bet+ cells (*P* < 0.05) [93]. The most important prognostic factor among these was found to be CD3 + TILs in the intra-tumoral (IT) compartment, with a pooled hazard ratio (HR) of 0.52 (95% CI 0.43–0.63; *P* < 0.001). Nevertheless, no significant correlation was seen between CD4 + TILs and survival outcomes. The prognostic effects of FOXP3 + TILs were interestingly bidirectional, with a beneficial impact on the extra-tumoral (ET) compartment (HR = 0.76; *P*-value = 0.022) and a negative impact on the intra-tumoral compartment (HR = 1.57; *P* = 0.033). The study concluded that some TIL subtypes might be predictive biomarkers for gastric cancer, offering important information about how they might be used to direct treatment plans [93].

Furthermore, to examine the prognostic and predictive utility of TILs in stage II–III GC patients after adjuvant chemotherapy, a post-hoc analysis of the CLASSIC trial was conducted by Liu et al. [94]. TIL density was measured from hematoxylin–eosin (HE) stained tumor sections in 629 patients from the CLASSIC trial and 307 patients from the Yonsei Cancer Center (YCC) using deep learning-based image processing. The findings demonstrated that when treated with surgery alone, GC patients with high TIL density had noticeably longer DFS than those with low TIL density. Furthermore, CLASSIC trial patients with low TIL density who had surgery and adjuvant chemotherapy had better DFS than those who underwent surgery alone. TIL density did not significantly correlate with any other clinicopathological factors. The study was sound, and this trial was at low risk of bias with a high sample size. According to the study, TIL density may be a clinically valuable biomarker for GC patients who might benefit from adjuvant chemotherapy; hence, it merits additional confirmation in prospective research [94].

#### Hepatocellular carcinoma

In a phase I trial that was conducted by Jiang et al., the TILs were isolated, activated, and expanded ex vivo before reinfusion in 15 HCC patients who received TIL therapy after tumor resection [95]. Of the 17 patients, 15 (88%) had effective TIL growth, and all 15 were still alive at the median follow-up of 14 months, with 12 (80%) exhibiting no evidence of disease [95]. Nevertheless, three individuals had tumor recurrences between days 105 and 261 post-therapy. With very minor adverse effects, such as flu-like symptoms, lethargy, leucopenia, and neutropenia, the medication was well tolerated. This study raised some concerns related to the confounding bias and low sample size, which are not representative of the whole HCC population [95]. Furthermore, the TIL infusion increased the number of CD8 + IFN-γ-producing T cells, indicating a stronger immune response against the tumor. According to the study’s findings, TIL-based immunotherapy is a safe and practical treatment option for patients with HCC, offering a potentially innovative approach with minimal toxicity and encouraging clinical outcomes [95].

#### Pancreatic cancer

TIL is a possible add-on therapy to standard treatment for pancreatic cancers. Regarding TIL presence (TIL + vs. TIL-) and PD-L1 expression (PD-L1 +), patients with locally recurrent pancreatic cancer treated with Stereotactic body radiation therapy (SBRT) + pembrolizumab + trametinib (SBRT + K + M) or SBRT + gemcitabine (SBRT + G) were categorized in the secondary analysis of a randomized phase II trial conducted by Zhu et al. [96]. The SBRT + K + M group’s TIL + patients had a significantly higher survival rate (OS: 17.2 vs. 12.7 months, *P*-value = 0.036; PFS: 10.5 vs. 7.1 months, *P*-value = 0.012), according to the results, whereas the SBRT + G group showed no change. Across subgroups, adverse occurrences were comparable [96]. With MEK inhibition and PD-1 blocking boosting TIL infiltration and increasing outcomes in pancreatic cancer, these results imply that PD-L1 expression and TIL presence may be biomarkers to predict response to radiation-immunotherapy combinations [96].

#### Colorectal cancer (CRC)

TILs regarding different locations of the tumor, as well as their impact on survival in stage III colon cancer, were also addressed [97]. Higher TIL concentrations in right-sided tumors than left-sided malignancies (*P*-value < 0.0001) were reported, based on a 1532 patients phase III trial analysis [97]. While low TIL levels had no discernible impact on left-sided tumors (*P* = 0.1731), they were associated with worse DFS in right-sided tumors (5-year DFS: 57% vs. 77%, HR = 2.02, *P*-value < 0.0001) [86]. Low TILs exclusively predicted worse DFS in right-sided tumors in low-risk patients (T1–3, N1), whereas low TILs were linked to worse DFS in high-risk patients (T4 and/or N2), irrespective of tumor site (*P*-value < 0.025) [97]. TILs are an important prognostic indicator since their contribution to DFS was much larger in right-sided tumors (24%) compared to left-sided tumors (1.5%), and it was 42% in high-risk tumors [97].

Another study suggested that although tumor-infiltrating CD8+ lymphocytes (TILs) have a predictive value that varies by tumor and nodal stage, they are linked to a decreased recurrence of CRC [98]. After verifying results in 1375 cases and analyzing 1804 stage II/III CRC cases from clinical trials, a lower recurrence risk corresponded with increased CD8+ density (HR = 0.92, *P*-value = 0.0036) was identified [98]. This effect, however, was most substantial in high-risk instances (HR = 0.87, *P* = 0.0094), moderate in intermediate-risk cases (HR = 0.92, *P* = 0.046), and nonexistent in low-risk (pT3, N0) cases (HR = 1.03, *P* = 0.75) [87]. These results imply that CD8+ TILs could improve CRC risk classification, especially for high-risk patients [98].

### Challenges and limitations

Several problems limit the broad use of TIL treatment in GI cancers:

- *Difficulty isolating TILs*: The production of efficient TIL cultures is complicated by the dense stromal environment of some tumors, such as pancreatic cancer, which prevents lymphocyte infiltration and subsequent isolation [99].
- *Immunosuppressive tumor microenvironment (TME)*: regulatory T cells, myeloid-derived suppressor cells, and immunosuppressive cytokines are frequently found in the TME of GI cancers. These factors can lower the therapeutic efficacy of infused TILs by inhibiting their activity [100]. The efficacy of the TILs could be inhibited by different factors within the TME, including regulatory T cells, myeloid-derived suppressor cells, and immunosuppressive cytokines [100].
- *Limited persistence of TILs in vivo*: TILs may exhibit limited persistence after reinfusion due to factors such as a lack of necessary cytokine support or inhibitory signals within the TME, leading to a transient therapeutic effect [101].

### Ongoing trials (Clinicaltrials.gov)

As of 2025, several clinical trials are actively investigating TIL therapy in GI malignancies, as shown in Table 3. These trials aim to optimize TIL expansion protocols, identify predictive biomarkers of response, and develop strategies to overcome the immunosuppressive TME, thereby enhancing the therapeutic potential of TIL therapy in GI cancers.

**Table 3:** Ongoing clinical trials investigating tumor-infiltrating lymphocyte (TIL) therapy in GI malignancies

NCT Number	Phases	Study status	GI malignancy	Intervention	Target	Primary outcome measures	Start date
NCT06532799	Phase I/II	Recruiting	Stomach and Esophageal cancer	Tumor-infiltrating lymphocytes (TIL) therapy combined with Pembrolizumab	PD-1 receptor on T cells	Objective response rate (ORR)	10/9/2024
NCT03935893	Phase II	Recruiting	Gastric/esophagogastric, colorectal, pancreatic	Autologous tumor-infiltrating lymphocytes (TIL) + high-dose aldesleukin	focuses on TIL therapy for GI malignancies	Objective response rate (ORR)	3/12/2019
NCT03935893	Phase II	Recruiting	Gastric/Esophagogastric, Colorectal, Pancreatic	Autologous tumor-infiltrating lymphocytes (TIL) therapy	Tumor-infiltrating lymphocytes assessed for potency (Interferon-gamma release)	Objective response rate (ORR)	3/12/2019
NCT03904537	Phase I/II	Unknown	Colorectal (Stage III colon cancer)	Anti-PD1 antibody-activated autologous tumor-infiltrating lymphocytes (TILs) + XELOX Chemotherapy	programmed cell death protein 1 (PD-1)	Safety and 3-year disease-free survival (DFS)	19/2/2019
NCT04426669	Phase I/II	Active, not recruiting	Metastatic gastrointestinal cancers	Tumor infiltrating lymphocytes (TIL) with CRISPR-edited CISH inhibition	CISH (Cytokine-induced SH2 protein)	Safety and efficacy of genetically engineered, neo-antigen-specific TILs	15/5/2020

## Overcoming challenges in GI malignancies

GI malignancies, encompassing cancers such as colorectal, gastric, pancreatic, and hepatocellular carcinoma, present significant therapeutic challenges. Advancements in immunotherapy, particularly CAR-T cell therapy and TIL therapy, have shown promise. However, their Efficacy is often limited by immunosuppressive TME, suboptimal T-cell design, and therapy-associated toxicities. Addressing these challenges is crucial for enhancing treatment outcomes in GI cancers.

### Tumor microenvironment (TME)

Strategies to modulate TME are essential for improving immunotherapy efficacy. TME in GI malignancies is characterized by a complex network of cellular and molecular components that promote tumor progression and suppress anti-tumor immune responses. Strategies to modulate this immunosuppressive environment are essential for improving immunotherapy efficacy. The first choice is combining these therapies with Checkpoint Inhibitors. ICIs have transformed cancer treatment by preventing inhibitory mechanisms that limit T-cell activation. ICIs and CAR T-cell treatment have demonstrated synergistic benefits, which may help overcome the immunosuppression of TME. Targeting PD-1/PD-L1 interactions, for example, has boosted anti-tumor responses by enhancing CAR T-cell activity within the TME [79]. In fact, PD-1 blockade showed promising therapeutic effects when combined with long-course chemoradiotherapy in locally advanced rectal cancer patients [102]. Additionally, patient response to therapy could be predicted by machine learning models based on changes in gut microbiomes observed during treatment [103]. Moreover, PD-1/PD-L1 blockade was shown to improve to some extent with the inhibition of PCSK9, a protease that regulates cholesterol metabolism, possibly by modulating the infiltration of the immune cells within the TME, thus increasing the quantity and enhancing the antineoplastic activity of T lymphocytes [104].

### Enhancing efficacy

Achieving long-lasting therapeutic responses in GI cancers requires optimizing the form and functionality of adoptive cell treatments:

- *Enhancing CAR-T cell design* is a possible solution to increase efficiency. Dual-targeting and armored CARs are the results of developments in CAR-T cell engineering. Dual-targeting CARs are made to identify many tumor antigens at once, which lowers the possibility that the tumor would escape because of antigen loss. To increase their longevity and usefulness within the TME, armored CARs are designed to express co-stimulatory ligands or release cytokines [105].
- *For TIL therapy to be effective*, TIL’s ex vivo expansion procedures and in vivo persistence must be improved. To increase their therapeutic potential, techniques have been investigated, including co-culturing TILs with supporting cytokines and putting lymphodepletion regimens in place before TIL injection [106]. Another solution is to incorporate it with Cytokines. The anti-tumor activity of T lymphocytes can be enhanced by administering cytokines, such as interleukin-12 (IL-12). By improving effector T cell invasion and function, IL-12 has been demonstrated to rewire the TME and create a milieu more conducive to immunotherapy [99].

### Reducing toxicity

It is essential to reduce the side effects of CAR T-cell therapy so that it can be used more widely in GI cancers:

- *Controlled activation and safety switches*: In the case of extreme toxicities, CAR-T cells can be selectively eliminated by incorporating safety mechanisms such as inducible suicide genes. Furthermore, temporal regulation of T-cell activity can be achieved by developing CARs with regulated activation regions, which reduces off-target effects [107].
- *Handling cytokine release syndrome (CRS)*: CRS is a frequent and potentially fatal side effect of CAR-T cell treatment. To decrease the severity of CRS, proactive treatment techniques are used, such as corticosteroids and anti-cytokine medications such as tocilizumab, an IL-6 receptor antagonist [108].

## Future directions

ACT started as TIL therapy in oncology, but the development of CLDN18.2 has sped up the development of CAR-T, which is the primary focus in this area of ACT for GI tumors. The discovery of new, ideal targets for CAR-T cell therapy is anticipated to broaden its scope. Multimodal strategies, including antibody–drug conjugates and bispecific antibodies, are also being actively investigated for the treatment of GI malignancies [109]. The future of ACT will likely depend on its ability to establish advantages over these emerging techniques. Notwithstanding its potential, ACT’s wider implementation is constrained by substantial logistical, social, and economic obstacles. The challenge of manufacturing g-cells and the need for a highly established healthcare infrastructure are two significant barriers to this therapy’s relatively low acceptance rates. It is expected that an off-the-shelf method utilizing autologous cell sources will be able to address these issues. TIL and CAR-T cell therapy improvements will provide new options with a good safety margin for GI malignancies treatment protocols.

## Conclusion

Gastrointestinal malignancies account for around 50% of all human cancers, which emphasizes the necessity of innovating treatment strategies. In early-phase trials, CAR T-cell treatments that target markers such as CLDN18.2, CEA, CD133, and NKG2D have demonstrated promising efficacy with controllable toxicity profiles. Notably, CD133-targeted CAR-T cells have produced stable disease or partial response in subgroups of patients with pancreatic cancer, whereas CLDN18.2-directed CAR-T cells have shown good disease control rates in gastric, pancreatic, and gastro-esophageal malignancies. TIL treatment is linked to better survival outcomes in some gastrointestinal cancer subtypes and has also demonstrated potential, especially in MSI-H/dMMR tumors.

From the standpoint of clinical translation, these findings support the idea that the creation of “universal” CAR T-cell platforms that target CLDN18.2 should be given top priority in order to expedite production and increase patient eligibility. Standardized amplification and expansion protocols must be used for TIL therapy to ensure scalability, repeatability, and constant therapeutic potency. In order to overcome the immunosuppressive tumor microenvironment and improve therapy persistence, ACT methods may be combined with immune checkpoint inhibitors, targeted treatments, or cytokine modulation (e.g., IL-12). Additionally, adding predictive biomarkers like PD-L1 expression or TIL density into patient selection may improve results and minimize needless exposure to expensive or intense treatments.

Overall, thorough phase II/III trials, standardized methodologies, and multi-center collaboration will be necessary to translate these immunotherapeutic advancements from trial settings into standard clinical practice. To improve survival and quality of life for patients with gastrointestinal cancers, CAR-T cell and TIL therapies can be included in standard care by focusing on the best possible targets, strong manufacturing standards, and logical combination methods.

## Data Availability

No primary data were used, all data used from published articles.

## Abbreviations

ACT: Adoptive cell therapy
ALL: Acute lymphoblastic leukemia
CAR-T cells: Chimeric antigen receptor T-cells
CRC: Colorectal cancer
CRCs: Colorectal cancers
CRS: Cytokine release syndrome
DFS: Disease-free survival
dMMR: Mismatch repair-deficient
EC: Esophageal cancer
EGFR: Epidermal growth factor receptor
ET: Extra-tumoral
FDA: US food and drug administration
GC: Gastric cancer
GI: Gastrointestinal
HCC: Hepatocellular carcinoma
HE: Hematoxylin–eosin
HR: Hazard ratio
ICIs: Immune checkpoint inhibitors
IL-2: Interleukin-2
IT: Intra-tumoral
MHC: Major histocompatibility complex
MSI-H: Microsatellite instability-high
NKG2D: Natural killer group 2 member D ligands
OS: Overall survival
SBRT: Stereotactic body radiation therapy
TAAs: Tumor-associated antigen
TCR-T: T-cell receptors
TILs: Tumor-infiltrating lymphocytes
TME: Tumor microenvironment
TSAs: Tumor-specific antigens
VEGF: Vascular endothelial growth factor
YCC: Yonsei cancer center
